# A Low-Cost iPhone-Assisted Augmented Reality Solution for the Localization of Intracranial Lesions

**DOI:** 10.1371/journal.pone.0159185

**Published:** 2016-07-25

**Authors:** YuanZheng Hou, LiChao Ma, RuYuan Zhu, XiaoLei Chen, Jun Zhang

**Affiliations:** 1 Department of Neurosurgery, PLA General Hospital Hainan Branch, Sanya, Hainan, China; 2 Department of Geriatric Endocrinology, PLA General Hospital, Beijing, China; 3 Department of Neurosurgery, PLA General Hospital, Beijng, China; Institute of Automation Chinese Academy of Sciences, CHINA

## Abstract

**Background:**

Precise location of intracranial lesions before surgery is important, but occasionally difficult. Modern navigation systems are very helpful, but expensive. A low-cost solution that could locate brain lesions and their surface projections in augmented reality would be beneficial. We used an iPhone to partially achieve this goal, and evaluated its accuracy and feasibility in a clinical neurosurgery setting.

**Methodology/Principal Findings:**

We located brain lesions in 35 patients, and using an iPhone, we depicted the lesion’s surface projection onto the skin of the head. To assess the accuracy of this method, we pasted computed tomography (CT) markers surrounding the depicted lesion boundaries on the skin onto 15 patients. CT scans were then performed with or without contrast enhancement. The deviations (D) between the CT markers and the actual lesion boundaries were measured. We found that 97.7% of the markers displayed a high accuracy level (D ≤ 5mm). In the remaining 20 patients, we compared our iPhone-based method with a frameless neuronavigation system. Four check points were chosen on the skin surrounding the depicted lesion boundaries, to assess the deviations between the two methods. The integrated offset was calculated according to the deviations at the four check points. We found that for the supratentorial lesions, the medial offset between these two methods was 2.90 mm and the maximum offset was 4.2 mm.

**Conclusions/Significance:**

This low-cost, image-based, iPhone-assisted, augmented reality solution is technically feasible, and helpful for the localization of some intracranial lesions, especially shallow supratentorial intracranial lesions of moderate size.

## Introduction

Precise localization of intracranial lesions before surgery is very important. This information is required for surgeons to select an appropriate surgical approach, to position the patient, and to tailor the incisions. Because the head is a complex three-dimensional (3D) structure that lacks surface anatomical landmarks, precise localization of an intracranial lesion and its surface projection according to two-dimensional (2D) magnetic resonance (MR) images is always difficult, especially when the lesion is small [1]. Modern neuro-navigation systems are very helpful because they provide real-time feedback in three imaging planes and 3D models of the brain [2, 3]. Furthermore, when these systems are interfaced with surgical microscopes equipped with projection systems, they can depict surface projections of brain lesions in augmented reality (AR) [4, 5]. AR is a technique in which computer graphics are overlaid on a video or an image of the real world. In the resulting image, both of the original images coexist as a single image, to enable visualization of the internal structures underneath the overlying tissues, providing a transparent view of the surgical anatomy. Using this approach, the surgical plan can be intuitively and precisely determined [2, 3]. These visualization technologies are improving the accuracy and safety of operations [2–5], and are becoming an important component of neurosurgery [2, 3], where they are mostly used for craniotomy positioning [6–8]. However, navigation systems are very expensive; therefore, their availability in developing regions is often very limited [9]. Moreover, the MR images used for diagnosis are not suitable for these image guidance systems. Instead, a thin-slice 3D MR volume with or without fiducials is required before surgery. Together with the application of surgical navigation, the cost of a single surgical navigation service is often unaffordable for patients in developing regions. A low-cost technique able to locate brain lesions and their surface projections in AR before surgery, and capable of using standard diagnostic MR images, would be beneficial, especially if the requirement for highly sophisticated and expensive navigation systems could be avoided. In this study, we adapted an iPhone (Apple Inc., Cupertino, CA, USA) to partially achieve this goal, and then evaluated its accuracy and feasibility in a clinical neurosurgery setting.

## Materials and Methods

### Ethics Statement

This study was approved by the Medical Science Ethics Committee of the General Hospital of the Chinese People's Liberation Army. Signed informed consent for the taking of photographs was provided by each patient or an appropriate family member. The individual depicted in this manuscript gave written informed consent (as outlined in the PLOS consent form) for publishing of the case details.

### Patients

Between January 2014 and May 2016, 35 patients who received surgery in our hospital were recruited to the study, and their brain lesions were localized using an iPhone and MR images. The lesions were localized by either the first or third author, and were allocated randomly. The borders of the lesion were determined using the following criteria: (1) for lesions with homogenous enhancement (such as meningioma, glioblastoma, part of a high-grade glioma), the border followed the edge of the enhanced part of the lesion; (2) for lesions with heterogeneous enhancement, the border was determined by the extent of abnormal signal in the T2 fluid-attenuated inversion recovery image (examples include low-grade glioma and cavernous angioma). Patients who harbored an intracranial lesion with a highly diffuse boundary and a border that was difficult to identify were excluded from the study.

### MR image preprocessing

**Fig 1:**
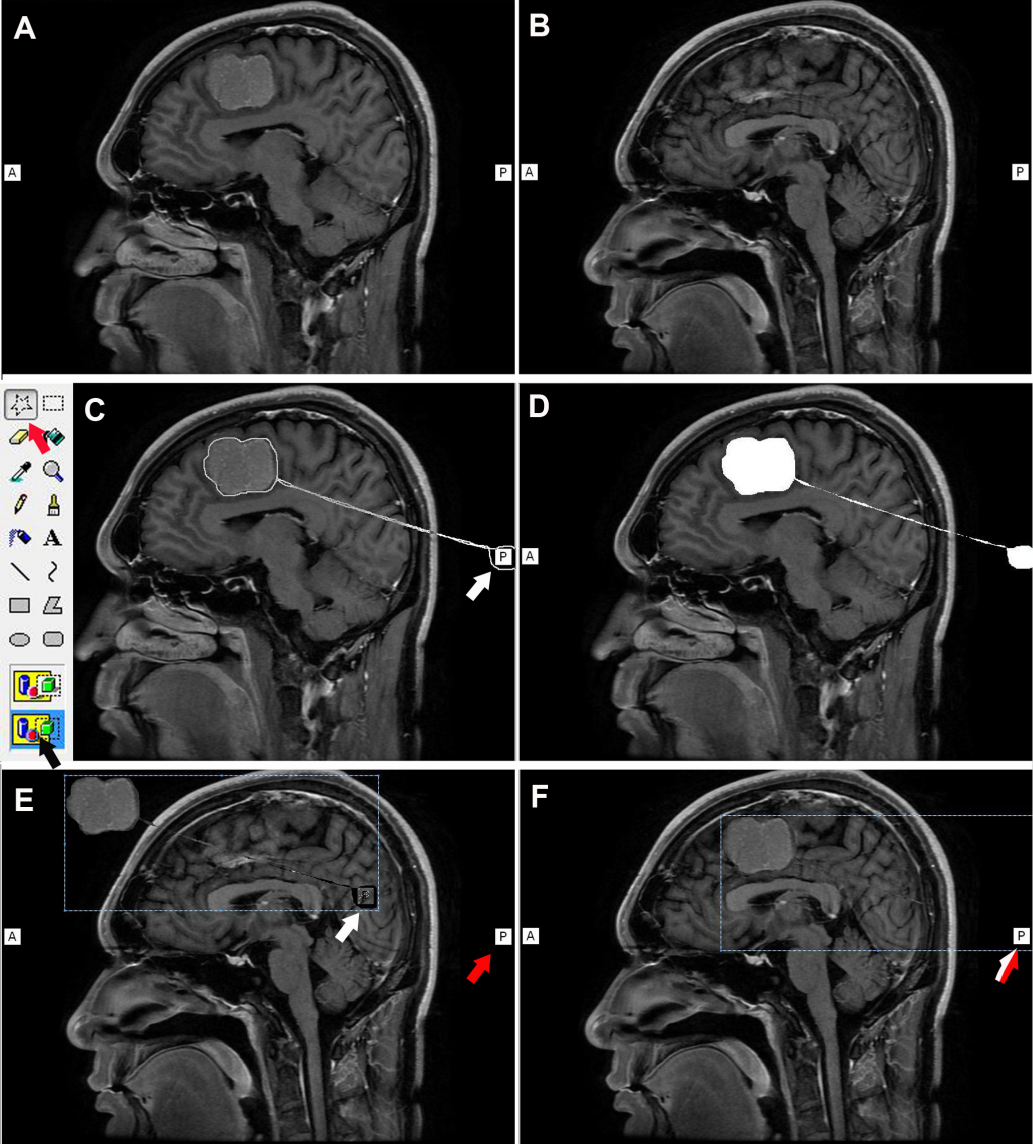
Preprocessing of the MR images. (A) A sagittal slice showing the maximal tumor boundary is selected as the first image. (B) A mid-sagittal slice is selected as the second image. (C) In MS Paint, the “Free-Form Select” tool (red arrow) and “Transparent Select” tool (black) are chosen. The tumor and the “P” label (white arrow) are selected together. (D) The selected tumor and the “P” label are cut out together. (E) The selected tumor and “P” label (white arrow) are pasted into the mid-sagittal slice. The red arrow indicates the corresponding “P” label in the mid-sagittal slice. (F) By making the two “P” labels overlap (half white and half red arrow), the projection of the tumor on the mid-sagittal slice is correctly depicted.

All MR images were accessed using the hospital’s picture archiving and communication system (PACS; Release 2.3; Philips Healthcare Informatics, Inc., Foster City, CA, USA). We used standard Windows XP image processing software (MS Paint; Microsoft Corporation, Redmond, WA, USA) to preprocess the MR images. The MR sequence type was selected on the basis of the surgical requirements and image characteristics of the lesion. If required, T2 weighted images or other MR sequences could also be used for this method. First, all of the sagittal slices were examined using the PACS, and the slice containing the maximal lesion boundary was selected and saved as the first image (Fig 1A). The mid-sagittal slice image was then selected and saved as the second image (Fig 1B). During this process, the magnification of the MR images was kept unchanged. Next, the first image was opened in MS Paint (Fig 1C), and the “Free-Form Select” (red arrow in Fig 1C) and “Transparent Select” (black arrow in Fig 1C) tools were chosen. The lesion was then circumscribed, together with the label “P” (white arrow in Fig 1C) corresponding to posterior orientation, and digitally cut out (Fig 1D). Following this, the second image was opened in MS Paint, and the tumor and label P from the first slice were pasted into it (white arrow in Fig 1E). The position of the pasted overlay was adjusted until the “P” label overlapped with its position in the first image (red arrow in Fig 1E). Because all of the selected sagittal slices belonged to the same sequence, they were all in the same coordinate system, and the position of the P label remained constant throughout all of the sagittal slices. Therefore, the projection of the tumor on the mid-sagittal slice could be correctly depicted by maintaining the overlapping of the “P” label between slices (red and white arrow in Fig 1F). Finally, the fused image was saved and transferred to an iPhone. All of these steps were completed within 5 min, without the need for additional software.

### Acquisition of the profile photograph of the patient

**Fig 2:**
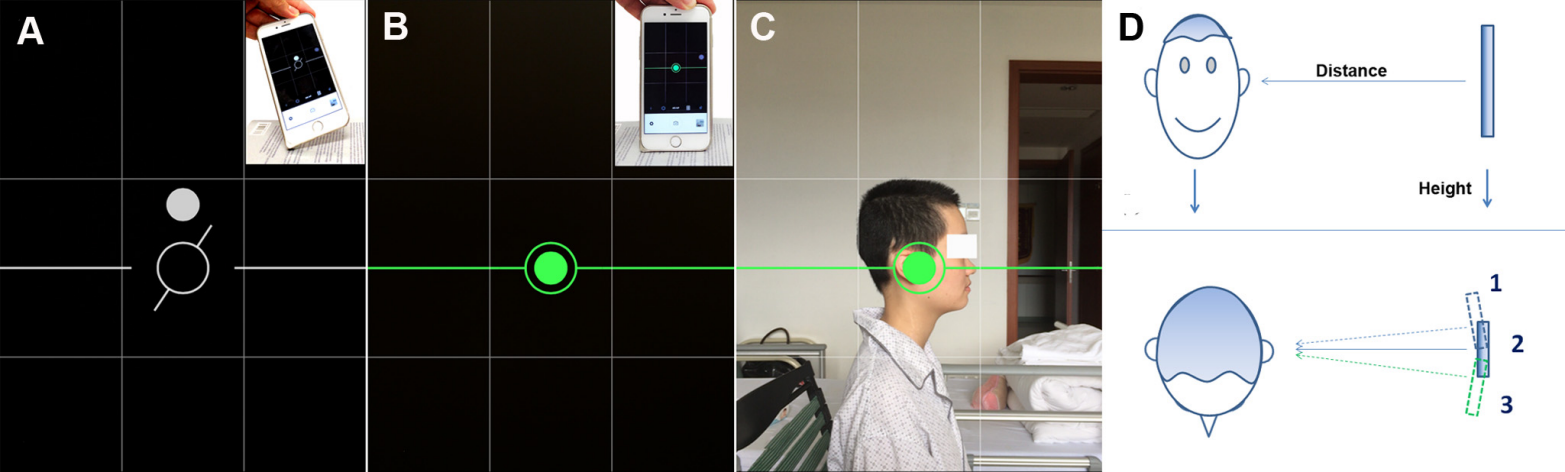
Acquisition of the sagittal photograph of the patient. (A) When the iPhone is tilted front-to-back, the white round dot deviates from the center of the circle in the LVL CAM iOS app (Daniel LLC, App Store; Apple Inc.). When the iPhone is tilted left-to-right, the short bar by the side of the round circle deviates from the horizontal line. (B) When the iPhone is vertical to the ground, the round spot and short bars turn to green, and the deviations are zero. (C) Aiming of the round marker at the external ear, and positioning of the patient’s head in the center square on the screen for acquisition of the photograph. (D) Illustration demonstrating the relative position between the iPhone and the patient’s head. In the frontal view, the patient’s head and the iPhone are both vertical to the ground. The distance and height are kept stable. In the view of the top of the head, fine changes to the shooting angle to find the best sagittal plane are demonstrated.

For acquisition of the patient’s sagittal photograph, the following techniques were adopted to eliminate angular mismatch between the sagittal photograph and the mid-sagittal MR image. First, the patients were asked to sit up straight without skewing or rotating their head. If the patient was unable to sit, they were asked to lie supine. An assistant stood in front of, or beside the patient, to ensure that the patient’s head position was correct. Second, LVL CAM (Daniel LLC, App Store; Apple Inc.) was used to take the photograph. The user interface (UI) of this iOS app is shown in Fig 2. If the iPhone was tilted (small picture in Fig 2A), the round spot would deviate from the circle at the screen center, and the short bar beside the circle would rotate away from the horizontal line (Fig 2A). Keeping the spot and short bars turned to green, indicating zero deviations (Fig 2B), would ensure that the iPhone was aligned vertically in all planes (small picture in Fig 2B). Third, the round marker was positioned over the external ear and the patient’s head was positioned within the center square on the screen (Fig 2C). Using these techniques, the relative position (distance and height) between the iPhone and patient’s head was standardized, as illustrated in Fig 2D. By finely adjusting the shooting angle, the best sagittal plane corresponding to position 2 in Fig 2D was then found.

### Co-registration of the MR images and sagittal photograph

**Fig 3:**
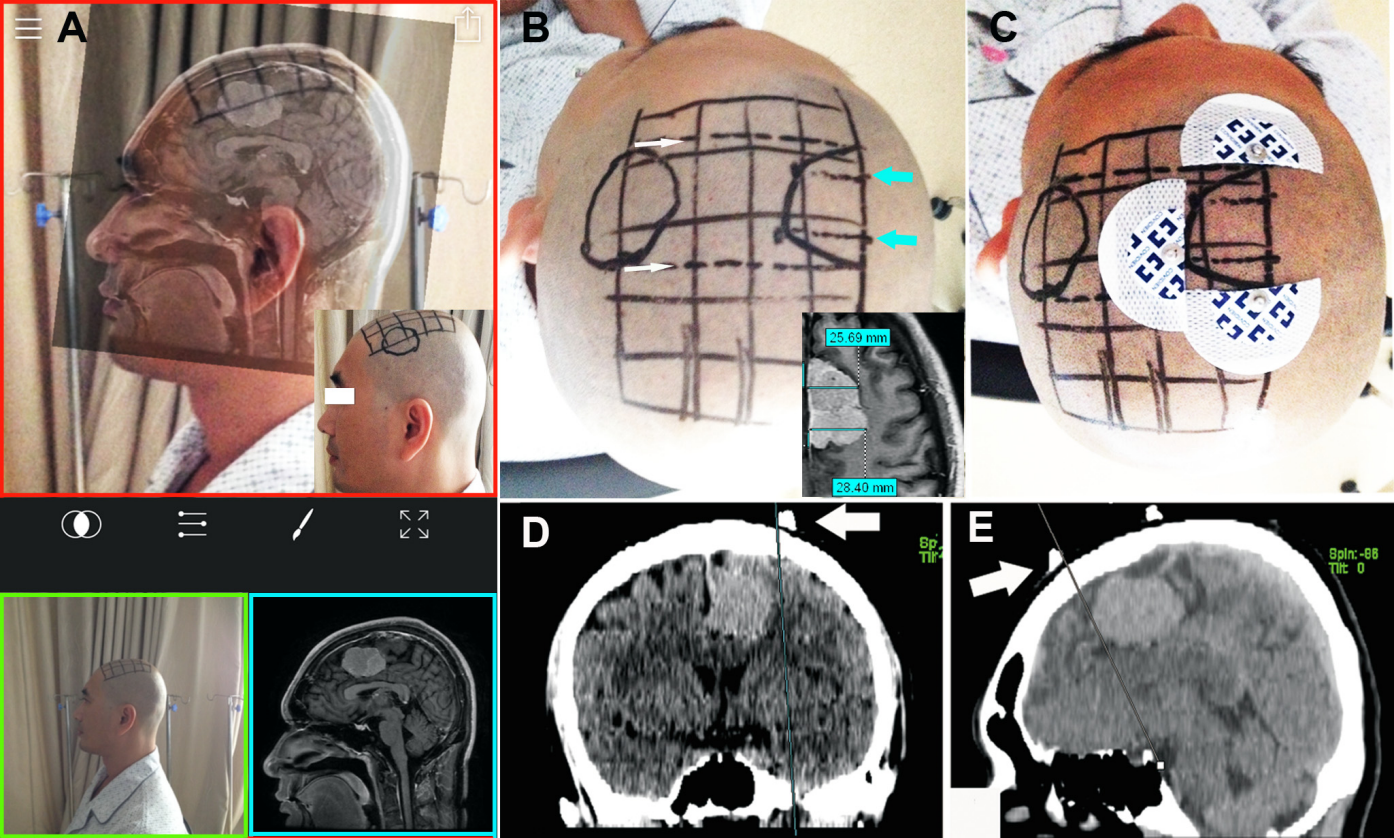
Localization of the lesion. (A) The user interface of FUSED (Easy Tiger Apps LLC; App Store; Apple Inc.). The sagittal photograph in the green panel was selected as the background and the MR image in the blue panel as the foreground. The two images are shown simultaneously in the red panel. Coregistration was performed according to anatomical landmarks. Gridlines are drawn on the skin in advance. With reference to the fused image, the lesion’s surface projection is depicted on the skin (small image in red panel). (B) By drawing two lines representing the vertical planes for the mid-sagittal plane (white arrows), the anterior and posterior tumor boundaries are determined. By measuring the distances of the lesion from the mid-sagittal line in an axial slice (small image), the lateral and medial boundary can be determined (blue arrows). (C) CT markers are pasted indicating the anterior, posterior, and lateral boundaries. (D) and (E) CT images with markers (white arrows). (D) On the coronal slice, a line touching the lateral edge of the lesion and the edge of the marker is drawn parallel to the midline. (E) On the sagittal slice, a vertical line touching the anterior edges of the lesion and the marker is drawn on the brain surface.

**Fig 4:**
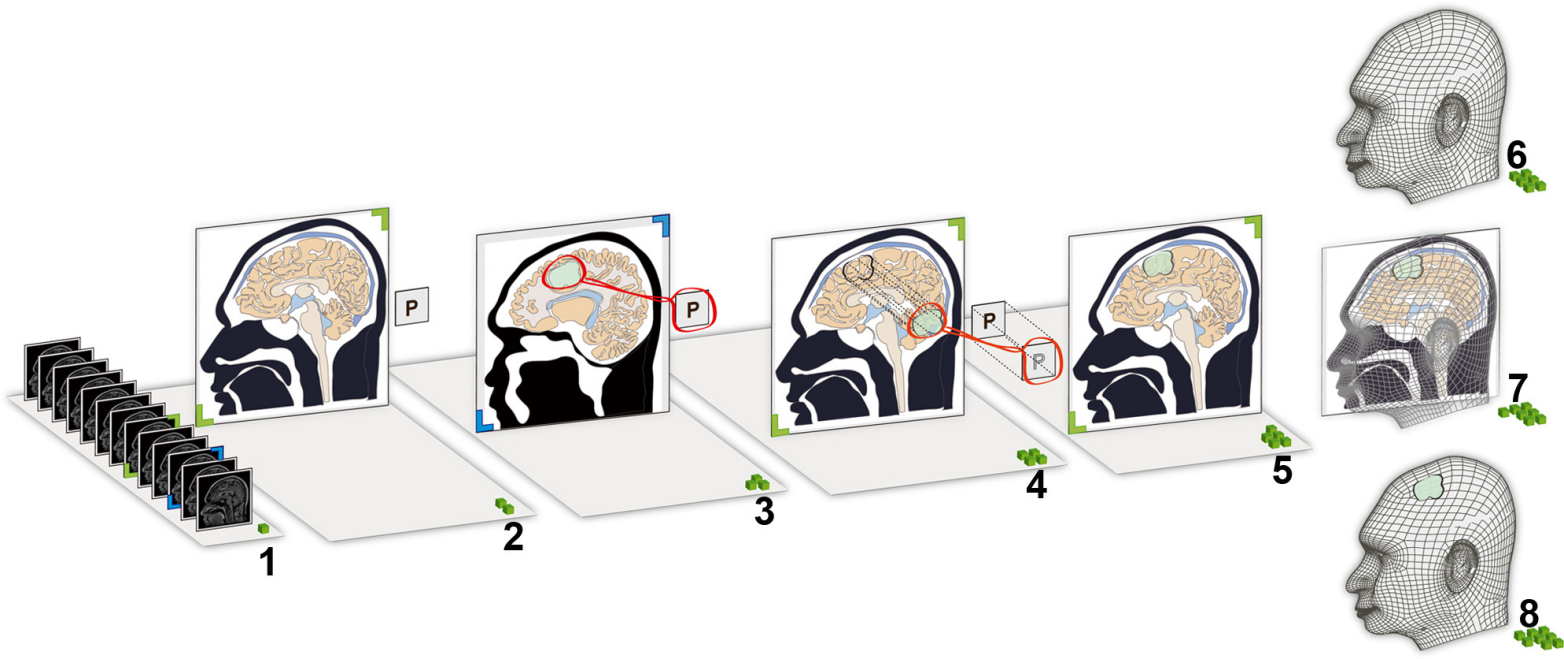
Schematic to present the method. 1, The sagittal MR images are browsed; 2, the mid-sagittal slice is selected; 3, the slice showing the maximal boundary of the lesion is selected. The tumor and the “P” label are cut out of the image together; 4, the tumor and the “P” label are overlaid on the mid-sagittal MR image by ensuring that the “P” labels overlap; 5, the tumor is correctly projected onto the mid-sagittal slice; 6, a sagittal photograph of the patient is obtained; 7, the mid-sagittal MR image is co-registered with the sagittal photograph of the patient. The lesion’s position is shown in an augment reality manner; 8, a surface projection of the lesion surface is depicted on the skin.

The FUSED app (Easy Tiger Apps LLC, App Store; Apple Inc.) was chosen to co-register the processed MR image to the sagittal photograph. The UI of this iOS app is shown in Fig 3A. The sagittal photograph was selected as the background (green panel in Fig 3A) and the MR image as the foreground (blue panel in Fig 3A). Thus, these two images could be shown simultaneously (red panel in Fig 3A). The transparency of the top MR image could be adjusted to ensure that the underlying photograph was also visible. The size and rotation angle of the top image were then manually adjusted, to precisely match the outline of the MR image with the outline of the patient’s head, keeping the following anatomical landmarks overlapping completely: lips, nasal tip, curve of head, and external occipital protuberance. Upon completion of the co-registration, the lesion’s location was clearly shown in AR (red panel of Fig 3A). Grids drawn onto the patient’s skin allowed the lesion’s sagittal projection to be depicted on the surface of the head (small image in red panel of Fig 3A). Fig 4 demonstrates the key stages in the process.

### Locating the axial projection of the lesion

After determination of the lesion’s sagittal projection, two further vertical lines of the mid-sagittal plane were drawn to represent the anterior and posterior boundaries of the lesion (the white arrows in Fig 3B). The distance of the lesion from the mid-sagittal line in the axial slice was then measured (small image in Fig 3B). This allowed the lateral and medial boundary to be determined (the blue arrows in Fig 3B). With reference to the two vertical lines and distances obtained from the axial slice, the lesion’s projection in the axial view could be depicted on the skin (Fig 3B).

### Verifying the accuracy of the new method

The accuracy of this new method was verified using two methods. In the first method (15 patients examined from June 2014 to December 2014), computed tomography (CT) markers were used to determine the accuracy. After depicting the lesion boundaries on the skin, two to four CT markers were pasted onto the surface of the head surrounding the lesion, to label the anterior and posterior and/or the medial and lateral boundaries (Fig 3C). The number of markers was selected according to the lesion location and size. If the lesion was adjacent to the midline, three markers indicating the anterior, posterior, and lateral boundaries were pasted. If the lesion was small (diameter < 1.5 cm), two markers to indicate the anterior and lateral boundaries were sufficient. These markers were actually electrode patches used for electrocardiographic monitoring. The metal portions of these patches had a radius of 4 mm, and could be clearly observed on the CT images. The patient then underwent CT scanning with or without contrast enhancement (Fig 3D and 3E). Using the PACS, we measured the deviation (D) between the lesion’s actual borders and the corresponding markers, to verify the accuracy of the markers. For a lesion in the frontal, parietal, or occipital lobe, the sagittal slices showing the anterior and/or posterior CT markers were selected first. The vertical lines of the sagittal contour of the head touching the lesion’s borders were drawn (Fig 3E). Next, the coronal slice showing the medial and/or lateral CT markers was selected. The lines touching the lesion’s medial and/or lateral borders were drawn parallel to the midline of brain (Fig 3D). The distances between these lines and the corresponding markers were measured and recorded as D. For a lesion in the temporal lobe or the basal ganglia, the axial slice was selected to verify the accuracy of the markers indicating the anterior and posterior borders of the lesion, and the coronal slice was selected to verify the accuracy of the markers indicating the top and bottom borders of the lesion. The accuracy levels of the markers were stratified into three groups, high (D ≤ 5 mm), moderate (5 mm < D ≤ 10 mm), and low level (D > 10 mm).

**Fig 5:**
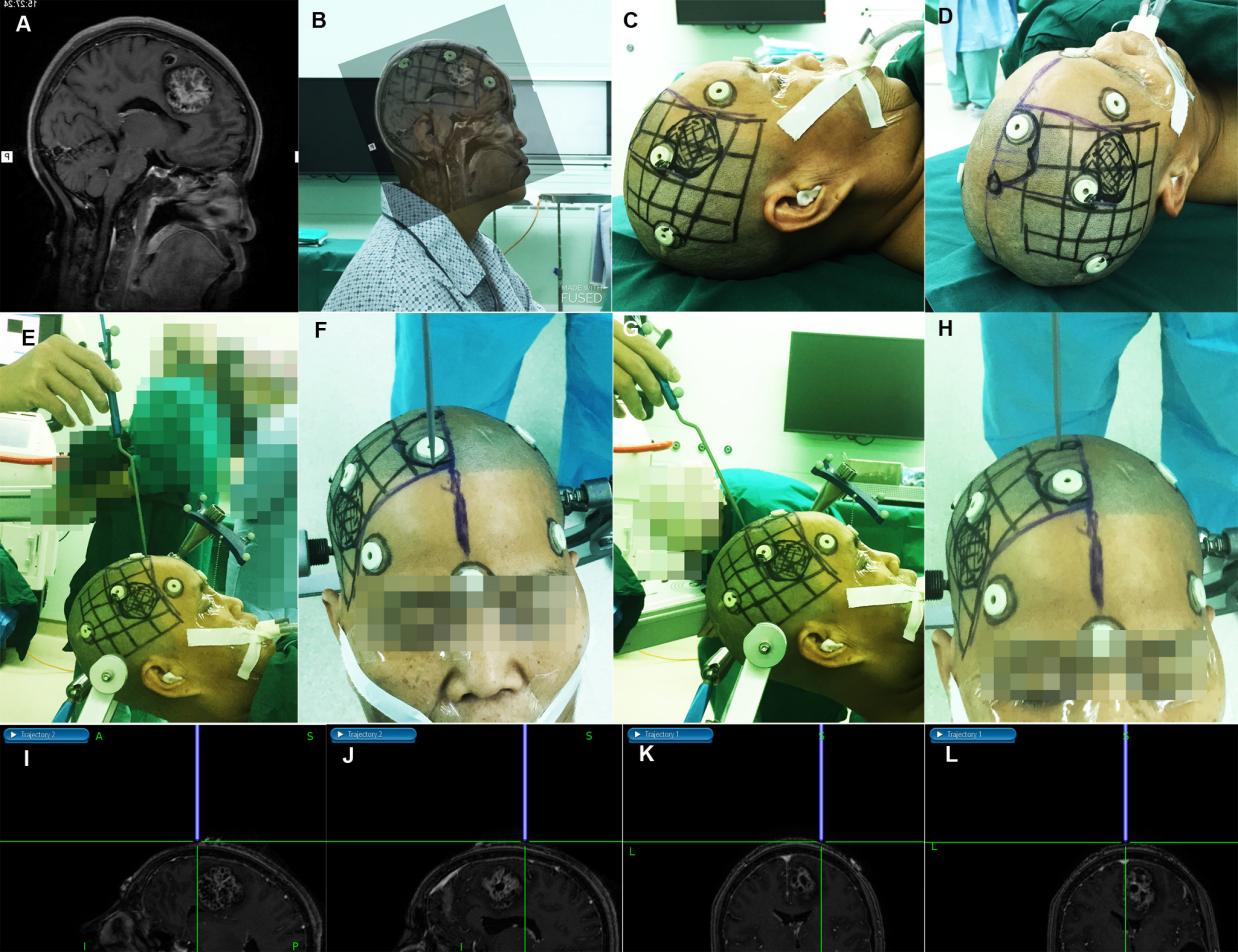
Comparing the new method with a frameless neuronavigation system. (A) Mid-sagittal T1 image of patient 6 in the second-stage trial, showing a cavernous angioma in the frontal lobe. (B) Co-registration of the mid-sagittal MR image with the sagittal photograph of the patient. (C) The lesion’s sagittal projection was marked on the skin according to the co-registration results. (D) In accord with the sagittal projection of the lesion, the axial projection was also depicted on the skin. (E) and (F) After anesthesia and co-registration with the navigation system, the operator pointed the navigation probe to the anterior boundary of the lesion. The probe direction was adjusted to be parallel with the sagittal plane and vertical to the mid-sagittal line. (G) and (H) The operator pointed the navigation probe to the posterior boundary of the lesion. (I) On the display screen, the “trajectory 2” navigation mode was chosen; The thick blue line indicated the navigation probe, which was vertical to the head surface. Its extended line, which touched the anterior boundary of the lesion, was green. The deviation was 0 mm. (J) The deviation at the lesion’s posterior boundary was also zero. (K) The “trajectory 1” model was chosen, and the deviation at the lesion’s lateral boundary was 2.3 mm. (L) The deviation at the lesion’s medial boundary was 0 mm.

In the second stage (20 patients from January 2015 to April 2015), we compared this method with the frameless neuronavigation system used in our hospital (Stealth Station S7; Medtronic Navigation, Louisville, KY, USA). Before surgery, six to eight markers were pasted onto the head. MRI scans were performed on a 1.5 Tesla scanner (Espree, Siemens, Erlangen, Germany) using a T1 weighted 3D MPRAGE sequence (TE 3.02 ms, TR 1650 ms, matrix size 256 × 256, FOV 250 × 250 mm, slice thickness 1 mm). The 3D dataset would be used by the navigation system. Before anesthesia, we depicted the lesions’ surface projections onto the skin using the new method (Fig 5C and 5D). After anesthesia, the surgeon correctly positioned and fixed the patients’ head and performed registration to the navigation system. The registration metric error, which was calculated by the navigation system to indicate the registration accuracy, was recorded. From the anterior, posterior, medial, lateral, superior, or inferior poles of each lesion’s surface projection, we chose four check points to verify the accuracy using a navigation probe (Passive Planar Probe; Medtronic Navigation; Fig 5E, 5F, 5G and 5H). The probe’s direction was adjusted to be parallel with the sagittal plane (Fig 5F and 5H), and vertical to the head surface (Fig 5E and 5G). The navigation system could automatically draw the extended line from the tip of the navigation probe when using the “trajectory 1” and “trajectory 2” navigation models (Fig 5I, 5J, 5K and 5L). The deviation (D) of the extended line from the lesion’s true borders was measured and recorded. Consistency measurements between the two methods were stratified into high (D ≤ 5 mm), moderate (5 mm < D ≤ 10 mm), and low consistency (D > 10 mm). The integrated offset vectors between the new method and navigation system were estimated by the following equations (only the absolute values of the deviations were used in the calculation).

$$\sqrt{{\left(\left(D_{Anterior}+D_{Posterior}\right)/2\right)}^{2}+{\left(\left(D_{Medial}+D_{Lateral}\right)/2\right)}^{2}}\;\text{or}$$

$$\sqrt{{\left(\left(D_{Anterior}+D_{Posterior}\right)/2\right)}^{2}+{\left(\left(D_{Superior}+D_{Inferior}\right)/2\right)}^{2}}.$$

### Statistical analysis

In each trial stage, the patients were divided into two subgroups according to the operator: the first author performed the procedure on group A, and the third author on group B. The deviations of the two subgroups were compared using a Mann–Whitney U test. The magnitudes of the offset vectors of the two subgroups were compared using a Mann–Whitney U test. The comparisons of lesion size, distance from the brain surface, and registration error metric, were performed using an independent samples t-test or a Mann–Whitney U test. Spearman rank correlation was performed to analyze the relationship between the magnitudes of offset vectors and lesion volumes or distance from the surface. Values are presented as mean ± standard deviation (SD), or median and first (Q1) and third quartile (Q3). All statistical analyses were performed using SPSS 11.0 software (SPSS Inc., Chicago, IL, USA). The threshold for statistical significance was set at P = 0.05.

## Results

### Results of the first stage

A total of 15 (seven female, eight male) patients aged from 19 to 73 years were enrolled in the first-stage accuracy trial (Table 1). The entire brain lesion location process was completed within 10 min for each subject (not including the accuracy verification process). In most patients, contrast-enhanced T1-weighted images (T1+C) were chosen for the location process, although sagittal T2-weighted images were used in two patients, and MR venography images were used for locating the transverse sigmoid sinus junction in one other patient. The lesion volumes ranged from 1.1 to 33.6 cm^3^ (15.1 ± 11.5 cm^3^). The median distance from the lesion to the brain surface was 5.8 mm (0, 9.0 mm), with 86.7% of the lesions (13 of 15) located within 10 mm of the brain surface. In eight patients, the lesions were localized by the first author, while the third author performed the localization process in the other patients.

**Table 1:** Patient characteristics in the first stage.

No	Gender	Age (years)	Lesion type	Location	Imaging modality	Size (cm^3^)	Distance to the brain surface (mm)	Operator (first or third author)
**1**	Male	49	Glioblastoma	Occipital lobe	T1+C*	24	5.8	First
**2**	Female	28	Cysticercosis	Posterior frontal lobe	T1+C	1.7	8.2	First
**3**	Female	56	Meningioma	Medial frontal lobe	T1+C	4.8	0	Third
**4**	Male	73	Cysticercosis	Posterior temporal lobe	T1+C	1.2	5.6	Third
**5**	Female	59	--	Transverse sigmoid sinus junction	MRV^#^	--	--	First
**6**	Male	55	Cyst	Basal ganglia	T2^##^	33.6	23.9	First
**7**	Male	30	Meningioma	Medial frontal lobe	T1+C	17.9	4.0	Third
**8**	Female	43	Meningioma	Medial frontal lobe	T1+C	2.7	0	First
**9**	Male	44	Glioblastoma	Posterior frontal lobe	T1+C	25	6.7	First
**10**	Female	24	Ganglioglioma	Posterior frontal lobe	T1+C	1.1	6.0	First
**11**	Male	19	Arachnoid cyst	Parietal lobe	T2	32	0	Third
**12**	Male	44	Abscess	Temporal lobe	T1+C	18	9.8	Third
**13**	Female	53	Meningioma	Occipital lobe	T1+C	20.9	0	First
**14**	Male	55	Hematoma	Basal ganglia	T1	18.6	21.5	Third
**15**	Female	46	Meningioma	Posterior frontal lobe	T1+C	9.4	0	Third

* Contrast enhanced T1 weighted images

# Magnetic resonance venography

## T2 weighted images

A total of 43 markers were created, and median deviation of these markers was 0 mm (0, 1.50 mm)(Table 2). For 97.7% of the markers (42 of 43), the accuracy level was high (deviation ≤ 5 mm), while it was rated as moderate for the other marker (2.3%, 1 of 43). Moreover, 74.4% of the markers (32 of 43) precisely depicted the lesion boundaries (deviation = 0 mm). Deviations from the true lesion borders were found for 18.6% of the markers (8 of 43), although in 87.5% of these (seven of eight), the deviation was below 4 mm. The maximum deviation measured was 6.5 mm. The lesions in 13 patients were accurately reached during surgery, even in patient number 2, who had a lesion measuring only 1.7 cm^3^. In patients 4 and 10, whose lesion volumes were only 1.2 and 1.1 cm^3^ respectively, the stereotactic frame was used to help in finding the lesions during surgery. In patient 5, the transverse sigmoid sinus junction was also accurately located using a marker, which was placed exactly over this anatomical landmark (deviation = 0 mm).

**Table 2:** Markers’ deviation and accuracy level.

No	Marker number	Marker location	Deviation (mm)	Accuracy
**1**	3	Anterior	0	High
		Lateral	0	High
		Posterior	3.5	High
**2**	3	Anterior	3.3	High
		Posterior	0	High
		Lateral	0	High
**3**	3	Anterior	0	High
		Posterior	0	High
		Lateral	1.5	High
**4**	2	Anterior	3.2	High
		Posterior	0	High
**5**	1	Top	0	High
**6**	4	Anterior	0	High
		Posterior	0	High
		Superior	0	High
		Inferior	0	High
**7**	3	Anterior	0	High
		Posterior	0	High
		Lateral	0	High
**8**	2	Anterior	0	High
		Posterior	2.4	High
**9**	4	Anterior	0	High
		Posterior	0	High
		Lateral	3.8	High
		Medial	0	High
**10**	2	Anterior	1.5	High
		Posterior	0	High
**11**	3	Anterior	0	High
		Posterior	0	High
		Lateral	2.8	High
**12**	3	Anterior	1.6	High
		Posterior	0	High
		Above	0	High
**13**	3	Anterior	0	High
		Posterior	0	High
		Lateral	0	High
**14**	4	Anterior	0	High
		Posterior	6.5	Moderate
		Superior	0	High
		Inferior	0	High
**15**	3	Anterior	0	High
		Posterior	2.0	High
		Lateral	0	High
**Total**	43		0(0, 1.50)	

In subgroup A, the median deviation of 21 markers was 0 mm (0, 0.38 mm), and in subgroup B, the median deviation of 22 markers was also 0 mm (0, 1.55 mm). There was no significant difference between the two subgroups (P = 0.75; Table 3). The other variables, including lesion size and distance to brain surface, were not significantly different.(Table 3).

**Table 3:** Comparison of markers’ deviation of different operators.

	Group A(8)	Group B(7)	p
**Marker’s number**	21	22	
**Deviation(mm)**	0 (0, 0.38)	0 (0, 1.55)	0.75
**Lesion’s volume(cm**^**3**^**)**	15.26±11.87	14.78±12.25	0.94
**Distance to the brainsurface(mm)**	6.00 (0, 8.20)	4.80 (0, 12.72)	0.73

### Results of the second stage

A total of 20 (five female, 15 male) patients aged from 4 to 67 years were enrolled in the second test of accuracy (Table 4). The lesion volumes ranged from 2.4 to 58.2 cm^3^.The distances from the lesion to the brain surface ranged from 0 to 49.3 mm, with 90% of the lesions (18 of 20) located within 10 mm of the brain surface. Ninety percent of the lesions (18 of 20) were supratentorial, and 10% (2 of 20) of the lesions were located in the cerebellum. In 11 patients, the lesions were localized by the first author, while the third author performed the pre-surgery localization in the remaining patients.

**Table 4:** Patient characteristics of the second stage.

No	Gender	Age (years)	Lesion type	Location	Size (cm^3^)	Distance to the brain surface (mm)	Operator (first or third author)
**1**	F	43	Meningioma	Frontal lobe parafalx	3.5	9.2	Third
**2**	M	18	metastase	Frontal lobe	58.2	7.1	First
**3**	M	51	Meningioma	Temperal lobe	9.4	0	First
**4**	M	21	Caverneous angioma	Frontal lobe	7.3	9.0	First
**5**	M	4	Choroid plexus papilloma	Third ventricle	2.4	49.3	Third
**6**	M	41	Caverneous angioma	Frontal lobe	15.5	5.3	Third
**7**	M	40	Meningioma	Frontal lobe	19.5	0	First
**8**	M	18	Metastase	cerebellum	6.9	8.0	Third
**9**	M	58	Meningioma	Parietal lobe parasigital sinus	10.8	0	First
**10**	F	46	Caverneous angioma	Temperal lobe	4.2	7.0	First
**11**	F	45	Arteriovenous malformation	Frontal lobe	44.7	0	Third
**12**	M	47	Gliablatoma	Occipital lobe	10.4	9.5	First
**13**	M	34	Meningioma	Parietal lobe	46.1	7.7	Third
**14**	M	67	Meningioma	Frontal lobe	6.2	0	First
**15**	M	42	Glioblatoma	Insula lobe	36.4	5	Third
**16**	M	62	Metastase	cerebellum	23.6	13.9	First
**17**	M	52	Meningioma	Frontal-parietal lobe	56.1	0	First
**18**	F	54	abscess	Frontal lobe	6.8	2	First
**19**	M	50	Caverneous angioma	Posterior frontal lobe	2.8	0	Third
**20**	F	22	Astrocytoma(II)	Frontal lobe	13.8	3	Third

For each patient, we checked the deviation at four points, resulting in a total of 80 check points (Table 5). The median deviation at these check points was 2.00 mm (0.00, 3.5 mm). High consistency between the new method and traditional neuronavigation system was found for 88.8% of the lesions. The magnitude of the integrated offset vector was 2.95 mm (2.43, 4.10 mm). In patient 12, who had an occipital glioblastoma, a modest deviation (> 5mm) was found at the superior and inferior boundaries. The registration error metric of 4.9 was the largest found in this group of patients. In patient number 11, who had giant arteriovenous malformations, the lateral edge of which were irregular and blurred in the MR images, a modest deviation of 9.5 mm was found at the lateral boundary. In two patients (8 and 16) with cerebellar metastases, the largest deviations (> 10 mm) were encountered at the superior and inferior boundaries. The registration error metrics in these two patients were greater than 2 (2.7 and 2.6). A Spearman correlation test demonstrated that the magnitude of the offset vectors did not correlate with lesion volume (P = 0.12) or distance from the brain surface (P = 0.75). For the supratentorial lesions, the median deviation of all 72 check points was 1.85 mm (0.00, 3.00 mm). The consistency between the new method and the navigation system at these check points was 93.1%, and the magnitude of the offset vector was 2.90 mm (2.35, 3.72 mm). A Spearman correlation test demonstrated that the magnitudes of the offset vectors did not correlate with lesion volume (P = 0.21) or distance from the brain surface (P = 0.20).

**Table 5:** Deviation between the results of new method and navigation system.

No	Registration error metric	Tumor’s boundaries	Deviation (mm)	Magnitude of offset vector
**1**	1.4	Anterior	3.5	3.2
		Posterior	2.8	
		medial	0	
		lateral	0	
**2**	1.5	Anterior	0	3.0
		Posterior	3.0	
		Superior	0	
		Inferior	5.2	
**3**	1.5	Anterior	4.0	3.8
		Posterior	0	
		Superior	2.0*	
		Inferior	4.5*	
**4**	1.9	Anterior	3.8	2.9
		Posterior	1.9	
		Medial	0	
		Lateral	0	
**5**	1.8	Anterior	0	1.5
		Posterior	2.5	
		Superior	0	
		Inferior	1.8	
**6**	1.5	Anterior	0	1.2
		Posterior	0	
		Medial	0	
		Lateral	2.3	
**7**	1.6	Anterior	2.5*	3.7
		Posterior	3.7*	
		Medial	0	
		Lateral	4.7	
**8**	2.7	Superior	11	12.1
		Inferior	13	
		Medial	0	
		Lateral	3.8	
**9**	1.9	Anterior	2.8	2.9
		Posterior	3.0	
		Medial	0	
		Lateral	0	
**10**	1.4	Anterior	1.5	2.4
		Posterior	0	
		Superior	0	
		Inferior	4.6	
**11**	1.6	Anterior	0	5.1
		Posterior	3.5	
		Medial	0	
		Lateral	9.5	
**12**	4.9	Superior	6.8	5.9
		Inferior	5.0	
		Medial	0	
		Lateral	0	
**13**	2.0	Anterior	2.4	3.0
		Posterior	3.6	
		Medial	0	
		Lateral	0	
**14**	1.8	Anterior	2.0	2.9
		Posterior	0	
		Medial	0	
		Lateral	5.5	
**15**	1.6	Anterior	0	2.5
		Posterior	4.3	
		Superior	2.6	
		Inferior	0	
**16**	2.6	Superior	12.5	14.2
		Inferior	15.8	
		Medial	0	
		Lateral	2.6	
**17**	1.8	Anterior	4.7*	4.2
		Posterior	3.6*	
		Medial	0	
		Lateral	0	
**18**	1.7	Anterior	2.5	2.6
		Posterior	2.3	
		Medial	2.3	
		Lateral	0	
**19**	1.5	Anterior	2.0	2.2
		Posterior	2.4	
		Medial	0	
		Lateral	0	
**20**	1.7	Anterior	0	1.9
		Posterior	3.0	
		Medial	0	
		Lateral	2.4	
**Total**	80		2.00(0.00, 3.58)	

* The direction of deviation was opposite.

In group A, where the operator was the first author, the deviation at 44 check points was 2.15 mm (0, 3.95 mm), and the magnitude of the offset vector was 3.00 mm (2.90, 4.20 mm). In group B, the deviation at the 36 check points was 0.90 mm (0, 2.95 mm), and the magnitude of the offset vector was 2.50 mm (1.70, 4.15 mm). There was no significant difference between the two subgroups (P *=* 0.3; Table 6). Other variables, including the lesion size, registration error metric, and distance from the brain surface, were not significantly different (Table 6).

**Table 6:** Comparison of deviation of different operators.

	Group A(11)	Group B(9)	p
**Check points**	44	36	
**Deviation(mm)**	2.15 (0, 3.95)	0.90 (0, 2.95)	0.30
**Magnitude of offset vector**	3.00 (2.90, 4.20)	2.50 (1.70, 4.15)	0.18
**Lesion’s size(cm**^**3**^**)**	10.40 (6.80, 23.60)	13.80 (3.15, 40.55)	0.71
**Distance to the brain surface(mm)**	2.00 (0.00, 9.00)	5.30 (1.50, 8.60)	0.50
**Registration error metric**	1.80 (1.50, 1.90)	1.60 (1.50, 1.90)	0.55

## Discussion

Precise localization of intracranial lesions is an extremely important, but difficult task, especially when lesions are small. A major cause of this difficulty is the fact that the brain is a complex 3D structure. The neurosurgeon must mentally transform the 2D MRI results into a 3D representation, and further imagine its 3D coordinates with respect to the patient’s brain [1]. To tailor the skin incisions and the bone windows, the surgeon must then transform the 3D coordinates of the lesion back into 2D surface projections, according to the patient’s position. These complex transformations are demanding, and require the surgeon to have experience and precise anatomical knowledge [1]. Another cause of localization difficulty is the shortage of surface anatomical landmarks that can be clearly identified on MR images. It is challenging to construct a real-world coordinate system to identify the location of an intracranial lesion according to anatomical landmarks. Image guidance technologies effectively solve this problem by performing reconstruction based on a 3D MRI volume and co-registering it with an accessional coordinate system [2, 3]. Furthermore, by displaying the location of intracranial lesions in AR, image guidance technologies can help specify very precise and intuitive surgical plans [4, 5].

Aside from the implementation of AR by image guidance systems and surgical microscopes, some authors have described alternative low-cost solutions [10–18]. These reports have confirmed the effectiveness of low-cost AR solutions in a neurosurgery setting. In these reports, the solutions for locating brain lesions are practical and do not require specially designed equipment. Lovo et al. described a solution in which a 3D reconstruction of the cerebral cortex and the venous circulation was co-registered with photographs of the patient’s head acquired using a digital camera [10]. The two images were then fused according to the fiducials attached to the patient’s head. The authors applied this method to eight patients and verified its accuracy using intraoperative ultrasound and frame-based stereotaxy. However, the authors did not provide data to evaluate the accuracy level. One limitation of the method was the requirement for an additional MRI scan with fiducials. Another point that was not addressed by the authors was how to maintain the consistency of the angle of the photograph and the 3D model-based digital image. This parameter is critical for performing accurate co-registration. In 2013, Mahvash et al. described an advanced AR solution, and demonstrated it using a phantom head [12]. In their study, they used a video projector to directly project a 3D model-based digital MR image onto the phantom’s head, skull, or brain surface in real time. Anatomical landmarks of the side of the head and five fiducial markers were used for manual registration. The final results were excellent; however, the authors did not provide an example of its clinical application. One limitation of this method is that manual adjustment of the focus, size, and position, of a projected virtual image, is a somewhat tedious process, especially when a patient is in a surgical position.

Owing to advancements in cameras, screens, and smart phone processors, AR can be implemented using mobile devices; methods using such devices have been referred to as mobile AR (mAR) [19]. This technology renders AR as much more convenient, affordable, and popular. Our solution of using an iPhone to assist in locating intracranial lesions, is a type of mobile AR implementation. Compared with the aforementioned AR solutions, our method achieved similar effects, but simplified the preprocessing of the MR images, the co-registration process, and the AR image generation. The method does not require additional cost or technical complexity, other than standard Windows XP image processing software (Paint), an iPhone, and a few iOS Apps. All of the processes could be completed within 10 min, without the need for a repeat MRI scan.

According to the results of the first stage, the deviation of 43 markers in 15 patients was 0 mm (0, 1.50 mm), and 97.7% of the markers displayed a high level of accuracy (D ≤ 5mm). Most deviations (87.5%) were smaller than 4 mm, with the maximum being 6.5 mm. Summing up these data, the maximum deviation of this new method should be around 5 mm. Mascott et al. described 30 surgical cases with frameless neuronavigation using the bilateral tragus, bilateral medial eye angle, and nasion for registration, and reported a deviation of approximately 5.4 mm [20]. Hence, the accuracy level of our technique should be close to their neuronavigation system. In the second stage of study, we further compared our method with a frameless neuronavigation system, and demonstrated a deviation between them of 2.95 mm (2.43, 4.10 mm). For the supratentorial lesions, the deviation was 2.90 mm [2.35, 3.72 mm]. The results were in the high consistency range for 93.1% of the 80 check points. If we further eliminate the effect of the single large registration metric error (patient 12), and irregular lesion edge (patient 11), the maximum offset was 4.2 mm. Summing up the registration error metrics of around 1–2 mm, the actual accuracy level of this new method should be around 5 mm, which is consistent with the results from the first stage. These data imply that when using anatomical landmarks for registration, our iPhone-assisted technique should have a similar accuracy level to the frameless navigation system.

**Fig 6:**
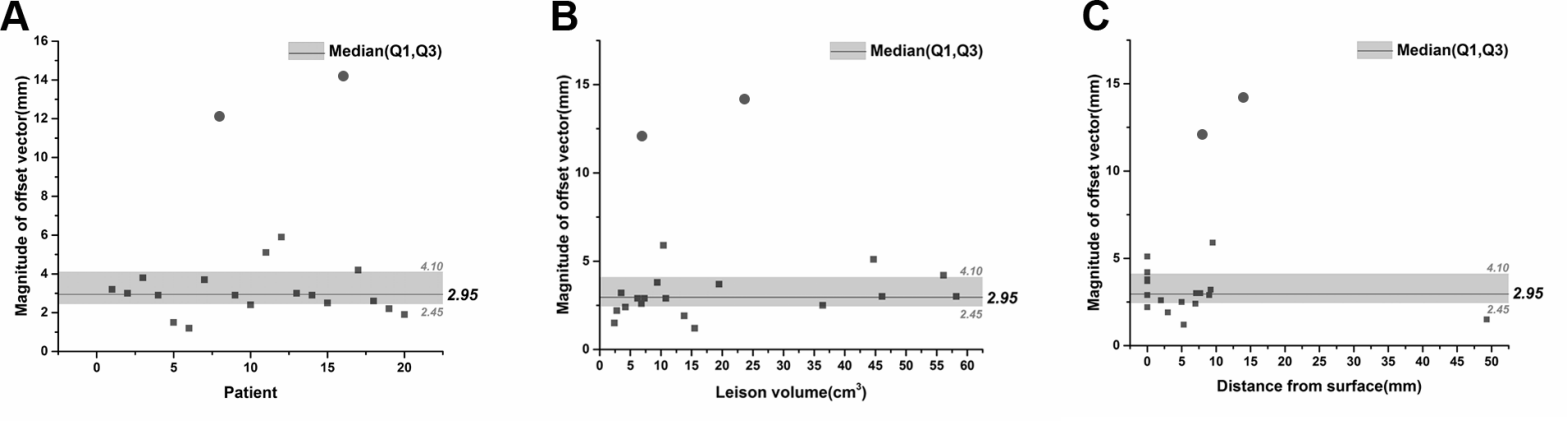
Magnitude of offset vectors in the second-stage trial. (A) Scatter plot graph illustrating the magnitude of offset vectors of the 20 patients in the second stage trial. The two round dots indicate the two patients with cerebellar lesions. The median, 1st and 3rd Quartile are labeled. (B) Scatter plot with the magnitude of offset vectors on the Y axis and the lesion volumes on the X axis. (C) Scatter plot with the magnitude of offset vectors on the Y axis and the lesion distance from the surface on the X axis.

We found that most of the variation in our method occurred in the acquisition of the sagittal photograph. Differences in camera height, distance from the patient, shooting angle, and zoom scale would result in differences to the photographs, which then led to further inconsistencies in the ensuing methods. The availability of techniques for controlling variations in the acquisition of the sagittal photograph is the reason why we chose the LVL CAM app, instead of the built-in iPhone camera app. In this study, we found no significant between-operator difference in the deviation data, in either of the test stages (Tables 3 and 6). In the second stage, we found that the magnitude of offsets for supratentorial lesions did not change with patient, tumor size, or distance from the brain surface (Fig 6). These findings indicate that other surgeons should be able to repeat this new method with acceptable accuracy levels.

The key step in our new method is the co-registration of the MR image with the sagittal photograph taken using the iPhone. We devised a simplified manual co-registration technique for this purpose. A similar technique was reported by Mahvash et al. [12]; however, unlike them, we did not rely on fiducials during co-registration. Our reasons for this are as follows; first, the profile of the human head and face is characteristic, and is easy to recognize. Anatomical landmarks on the surface of the skin, such as the nasion, anterior nasal spine, medial and lateral angles of the ipsilateral eye, ipsilateral tragus, and ipsilateral ear helix, were all confirmed to be effective for navigation registration [21]. Second, our technique using the LVL CAM app meant that the sagittal photographs could be easily acquired with the correct head angle, which could otherwise influence the silhouette of the photograph. Third, the mid-sagittal MR image slice was easy to obtain, and clearly showed the distinct outlines of the face and head. Furthermore, the co-registration can be performed on the touch screen, in an intuitive manner. Our data confirmed that this co-registration technique was feasible, and could be rapidly accomplished, even though it is currently a manual process. However, further developments of this methodology would be beneficial, and should include an automatic 2D/3D registration algorithm, an integrated and cross-platform mobile-app, and real-time augmented reality display technology.

According to our results, the application range of this technique would be as follows; first, this technique is not suitable for sub-occipital lesions. At the second stage, obvious deviations (>10 mm) were encountered in two patients with cerebellar metastases (patient 8 and patient 12). The main reason for these deviations was the substantial thickness of the sub-occipital muscle groups. To expose the cerebellum, the surgeon must bend the neck as far forward as possible. During this process, the surface projection of the lesion depicted on the skin would be stretched, and therefore deviate greatly from its original position. Second, this technique is most suitable for shallow lesions, which are partially exposed at the brain surface, or within 10 mm of the surface. The minimum lesion diameter should be larger than 2 cm if the lesion is not directly exposed at the surface of the brain. Eighty percent of the lesions in the first stage trials, and 90% of the lesions in the second stage, were of this type. We did not encounter any difficulties in finding these lesions during surgery. For very small intraparenchymal lesions (maximum diameter < 1.5 cm, such as in patients 4 and 10 in the first-stage trials), we had to use a stereotactic frame during surgery, to help with the location of the lesions. For very deep lesions, such as in patient 5 in the second-stage trials, a full navigation system had to be used. This technique is well suited for the puncture, intubation, and drainage surgery of deep-seated hematomas or brain abscesses, such as in patients 6, 12, and 14 in the first stage. According to the surface projection depicted on the skin, the site, direction, and depth for the puncture can be determined intuitively. The direction could also be easily adjusted and maintained during surgery. This allowed these three lesions to be accurately reached, even though they were deep seated (distance > 2 cm).

There are some limitations to this study. First, this was a single-center study with small sample sizes; multi-center studies with larger sample sizes are required to assess the feasibility and clinical impact of this new method. Second, we did encounter an inconvenient situation in clinical practice. Some patients had recently received MRI scans in other hospitals, and came to our hospital with their MRI films. We did not ask them to repeat the MRI scans, as these films were of good quality; therefore, the digital MRI images could not be attained through our PACS. Our alternative method was to put the MRI films, with all the sagittal slices, onto a film viewer and then photograph them with the iPhone. To reduce distortion of the photographs, we used LVL CAM to keep the iPhone parallel to the MRI film. These photographs of the MRI films could then be used for co-registration with the patients’ head photographs. These patients (currently numbering 3) were not included in the study because the number was too low. Taking photographs of the MRI films may introduce new errors into the results; therefore, the accuracy level in this situation still requires further investigation.

## Conclusions

This low-cost, image-based, iPhone-assisted AR solution is technically feasible, and helpful for the localization of some intracranial lesions, especially shallow supratentorial intracranial lesions of a moderate size.
